# Low-Cost and High-Performance ZnO Nanoclusters Gas Sensor Based on New-Type FTO Electrode for the Low-Concentration H_2_S Gas Detection

**DOI:** 10.3390/nano9030435

**Published:** 2019-03-15

**Authors:** Guodong Zhao, Jingyue Xuan, Xiaolin Liu, Fuchao Jia, Yuping Sun, Meiling Sun, Guangchao Yin, Bo Liu

**Affiliations:** Laboratory of Functional Molecular and Materials, School of Physics and Optoelectronic Engineering, Shandong University of Technology, Zibo 255000, China; gdzhaoo@163.com (G.Z.); xuanjingyue1124@163.com (J.X.); liuxiaolin0987@163.com (X.L.); fcjia515178@163.com (F.J.); sunyuping821008@163.com (Y.S.); sunml@sdut.edu.cn (M.S.)

**Keywords:** ZnO nanoclusters, etched fluorine-doped tin dioxide glass, low-concentration H_2_S, ohmic contact, cross-linked junctions, grain boundary barrier

## Abstract

A low-cost and high-performance gas sensor was fabricated by the in-situ growing of ZnO nanoclusters (NCs) arrays on the etched fluorine-doped tin dioxide (FTO) glass via a facile dip-coating and hydrothermal method. Etched FTO glass was used as a new-type gas-sensing electrode due to its advantages of being low cost and having excellent thermal and chemical stability. ZnO NCs are composed of multiple ZnO nanorods and can provide adequate lateral contacts to constitute the paths required for the gas-sensing tests simultaneously, which can provide many advantageous point junctions for the detection of low-concentration gases. The gas-sensing tests indicate that the ZnO NCs gas sensor has good selectivity and a high response for the low-concentration H_2_S gas. The sensing response has reached 3.3 for 500 ppb H_2_S at 330 °C. The excellent gas-sensing performances should be attributed to the large specific surface area of in-situ grown ZnO NCs, the perfect ohmic contact between ZnO NCs and FTO electrode and the variation of grain boundary barrier at the cross-linked junctions of multiple nanorods. In addition, the detailed effect of work temperature and gas concentration on gas-sensing, the stability of gas sensors and the corresponding response mechanism are also discussed in the present paper.

## 1. Introduction

Zinc oxide (ZnO), one of the most important metal-oxide-semiconductor materials, has been of great interest for gas sensor applications due to its peculiar characteristics such as nontoxicity, good chemical and thermal stability, the high mobility of conduction electron and low production cost [1,2,3,4,5,6]. It is well-known that the gas-sensing properties of ZnO strongly depend on its shape and dimensionality. Therefore, various structures of ZnO nanomaterials (zero-dimensional (0D) nanoparticles, 1D nanorods, 2D nanosheets, 3D hierarchical films) have been extensively studied [7,8,9,10,11]. Among them, 1D ZnO nanostructures have been regarded as one of the promising choices due to their unique advantages. For example, the large surface area can absorb more target gas molecules on the surface, the high aspect ratio can provide a direct and fast electronic transmission path and the facile preparation can reduce the cost of the experiment, and so on [12,13,14,15].

On the basis of 1D ZnO nanomaterials, the device structure of the gas sensor plays a vital role in the gas-sensing performances including the sensitivity, response, recovery and stability. For a typical gas-sensing device, the gas-sensing materials generally are coated on the prefabricated ceramics plate’s electrodes by a screen-printing or doctor-blading method, then annealed at an appropriate temperature to form a film for the gas-sensing detection [16,17]. However, it will inevitably lead to several drawbacks and deteriorate the device’s performance. For instance, the agglomeration of gas-sensing materials will make 1D ZnO nanomaterials lose the high surface-to-volume ratios and, as a result, only a small portion of gas-sensing materials can be exposed to the gas atmosphere for reacting with the target gas [18]. Also, the nonuniformity and breakage of films may influence the repeatability of batch preparation. In order to overcome these problems, many different structured gas sensors have been designed and investigated, such as the fabrication of gas sensors with the individual wire or rod as a sensing material [19], integrating field effect transistor (FET) components to obtain an amplified signal [20,21]. However, these devices require time-consuming and expensive experimental costs, which significantly limit their practical applications. Herein, a novel, low-cost, and stable gas sensor was designed and fabricated by the in-situ growing of ZnO NCs arrays on the etched FTO glass via a facile dip-coating and hydrothermal method in present work. Etched FTO glass is used as a new-type gas-sensing electrode due to its advantages of being low cost, having excellent thermal stability and being corrosion resistant. The necessary open-circuit structure for gas-sensing tests is obtained by a laser ablation technique. Uniform ZnO NCs arrays can construct the adequate self-assembled pathways with FTO electrode by the lateral connection of multiple ZnO nanorods for the gas-sensing detection, and which will maximize the surface area exposed to the test atmosphere and provide many advantageous cross-linked point junctions. Based on this unique structure, the ZnO NCs gas sensor should exhibit a fast response, with high sensitivity and good stability for the low-concentration H_2_S gas. The detailed effect of work temperature and gas concentration on gas-sensing, the stability of gas sensors and the corresponding response mechanism are also discussed in the present paper.

## 2. Materials and Methods

### 2.1. Materials

All reagents are of analytical grade and used without any further purification. Zinc nitrate hexahydrate, zinc acetate dehydrate were purchased from Sinopharm Chemical Reagent Co., Ltd. (Shanghai, China). Polyethylenimine (MW = 600) was purchased from Maclin Reagent Co., Ltd. (Shanghai China). The interdigital gas-sensing electrode was made of FTO glass (14 Ω/square). The FTO glass was etched in advance by a facile laser ablation technique to remove several strips of FTO conductive layer and form an interdigital pattern, in which the distance between adjacent fingers is 30 μm and the etched depth is 500 nm (equal to the thickness of FTO conductive layer).

### 2.2. Fabrication of the ZnO NCs Gas Sensor

Etched FTO electrodes (1.5 cm × 1.5 cm) were cleaned ultrasonically with dilute hydrochloric acid solution, acetone, isopropanol, ethanol, and deionized water for 30 min in turn, respectively, and subsequently dried with nitrogen before the growth of ZnO NCs arrays.

ZnO NCs arrays were prepared by a facile hydrothermal method with the assistance of the seed layer via a dip-coating method. Specifically, the ZnO seed solution was previously prepared by adding 0.05 M zinc acetate and 0.05 M diethanolamine to 40 mL absolute ethanol. Then using a self-made motor, the FTO electrode was dipped into the solution at a constant rate of 1 mm/s and then lifted off the solution at the same rate when the FTO electrode had all entered the solution. Herein, the FTO electrode and its long-etched strip were along the vertical direction to the solution. Next, the FTO electrode was dried overnight and then calcined for 10 min at 400 °C in a muffle furnace. The above procedure is repeated twice to successfully prepare the ZnO seed layer. For the growth of ZnO NCs arrays, 40 mL of aqueous solution that consisted of 0.05 M zinc nitrate hexahydrate, 0.055 M hexamethylenetetramine and 0.44 g polyethyleneimine were transferred to Teflon autoclave, while the FTO electrode was slightly tilted and placed at the bottom of it. Then, this system was maintained at 95 °C for 6 h to obtain in-situ ZnO NCs arrays on the FTO electrode. The fabricated samples were rinsed with deionized water and ethanol several times, and then were dried at 60 °C for 2 h in a drying oven.

### 2.3. Characterization and Gas-Sensing Measurement

The morphology of samples was analyzed by field emission scanning electron microscope (FESEM, SU8010, Hitachi, Tokyo, Japan). The crystalline phase of samples was identified by X-ray power diffraction (XRD, Germany Bruker AXS D8 Advance, Karlsruhe, Germany) with Cu-Kα radiation (λ = 1.5418 Å). The microstructure of samples was characterized by high-resolution transmission electron microscopy (HRTEM, JEOL JEM-2200FS, Tokyo, Japan). *I-V* characteristics were measured in an air atmosphere under room temperature (25 °C) and 330 °C by a source-measure system (Keithley 2400, Beaverton, OR, USA).

**Figure 1:**
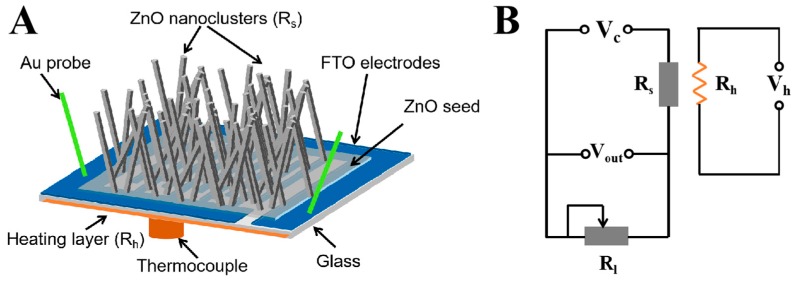
(**A**) The schematics of the ZnO NCs gas sensor and (**B**) the electric circuit of the sensor analysis system.

The electrical and gas-sensing properties were carried out using an intelligent gas-sensing analysis system (CGS-4TPS, Ltd., Beijing, China). The schematics of the ZnO NCs gas sensor and the electric circuit of the sensor analysis system are shown in Figure 1. V_c_ is a constant voltage power supply of 9 V, and V_out_ is an output voltage. R_l_ is the load resistance and R_s_ is the resistance of the sensor, and a heating voltage (V_h_) is used to adjust the working temperature. The sensor signal (S) of gas sensors is defined as the ratio of the resistance of ZnO NCs sensor before and after exposing them to target gas: S = R_a_/R_g_, where R_a_ is the resistance of gas sensor exposed to the air and R_g_ is the resistance exposed to the target gas atmosphere.

## 3. Results and Discussion

**Figure 2:**
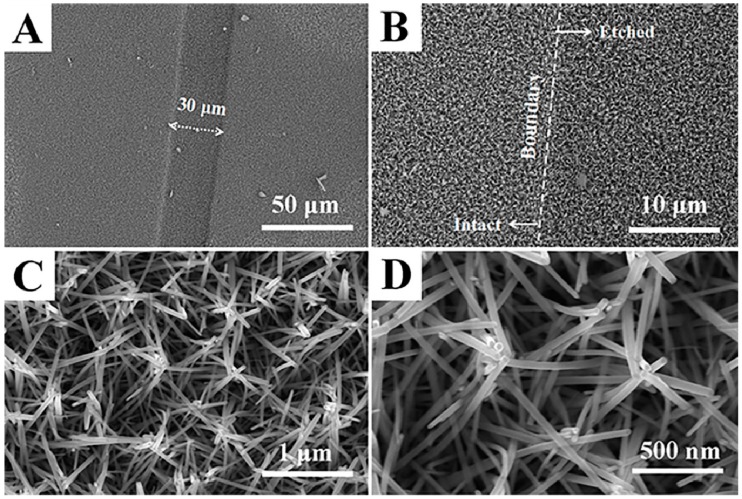
SEM images of ZnO NCs arrays at (**A**) 1000×, (**B**) 5000×, (**C**) 50,000× and (**D**) 100,000× magnification.

Figure 2 shows the SEM images with different magnifications of ZnO NCs arrays grown on the etched FTO electrode. As shown in Figure 2A, a strip-shaped trench with the width of 30 μm can be clearly seen, which corresponds to the etched strip formed by the laser ablation of the conductive layer of FTO glass. At the boundary of the etched strip (Figure 2B), ZnO NCs arrays grow along a small slope and have the same morphology at the boundary and on both sides. This means that the FTO conductive layer has good contact with the ZnO NCs arrays grown on the etched stripes, which plays a vital role in the formation of test pathways by nanobridges assembled by multiple ZnO nanorods. Moreover, the good uniformity can be seen at this magnification. For the details of ZnO NCs arrays (Figure 2C,D), the majority of the rod-shaped ZnO grew obliquely and a small fraction looked bent. As a result, several ZnO nanorods are interlaced with each other and contact each other at the top end, eventually forming a clustered ZnO array. Remarkably, this clustered morphology with a cross-linked structure can allow electrons to transfer between them and form a pathway to reach the electrode. Compared to the gas sensors fabricated by the doctor-blading method, this cross-linked structure can maximize the exposure of surface area to the gas atmosphere, because the loss of specific surface area due to lateral stacking and the agglomeration of nanorods could be effectively avoided. Furthermore, the proper distance between the nanorods can be observed, which will facilitate the diffusion of the target gas.

**Figure 3:**
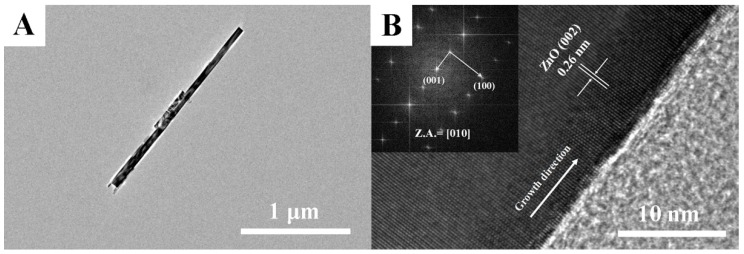
(**A**) TEM and (**B**) high-resolution (HR)TEM images of individual ZnO nanorod.

To further investigate the microstructure of ZnO NCs, the TEM and HRTEM examinations were performed. As shown in Figure 3A (TEM image), an individual ZnO nanorod with a length of 1.5 μm and a diameter of 50 nm is observed, which implies it possesses a high aspect ratio for more oxygen species adsorption sites. In the further HRTEM image (Figure 3B), the measured lattice spacing of 0.26 nm can be matched with (002) facet of wurtzite ZnO, which indicates that the ZnO nanorod grows along the [002] direction (*c*-axis). Moreover, the SAED pattern shown in the inset of Figure 3B indicates that ZnO nanorod is a single crystalline in nature without any impurity phase.

**Figure 4:**
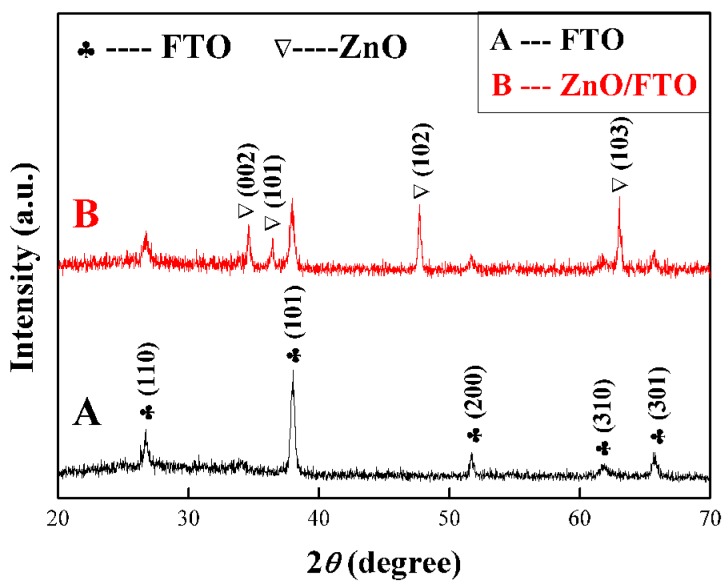
XRD patterns of (**A**) the etched FTO glass and (**B**) ZnO NCs array on the etched FTO glass.

The crystal structure of as-prepared samples is studied by XRD analysis, as shown in Figure 4. The FTO electrode was first tested as a reference (curve A), which displays the main diffraction peaks at 26.5°, 37.7°, 51.7°, 61.7° and 65.7°, corresponding to the tetragonal SnO_2_ (JCPDS no. 46-1088). After the in-situ growth of ZnO NCs arrays (curve B), four new diffraction peaks appear at 34.4°, 36.2°, 47.5° and 62.8°, respectively, which can match with the (002), (101), (102) and (103) facets of hexagonal ZnO (JCPDS no. 36-1451), indicating as-prepared ZnO NCs have a hexagonal wurtzite structure. Moreover, it is noteworthy that both (102) and (103) diffraction peaks exhibit a greater diffraction intensity than the (002) diffraction peak, which implies that most of ZnO nanorods are grown obliquely rather than vertically [15], which is consistent with the results of the SEM images.

**Figure 5:**
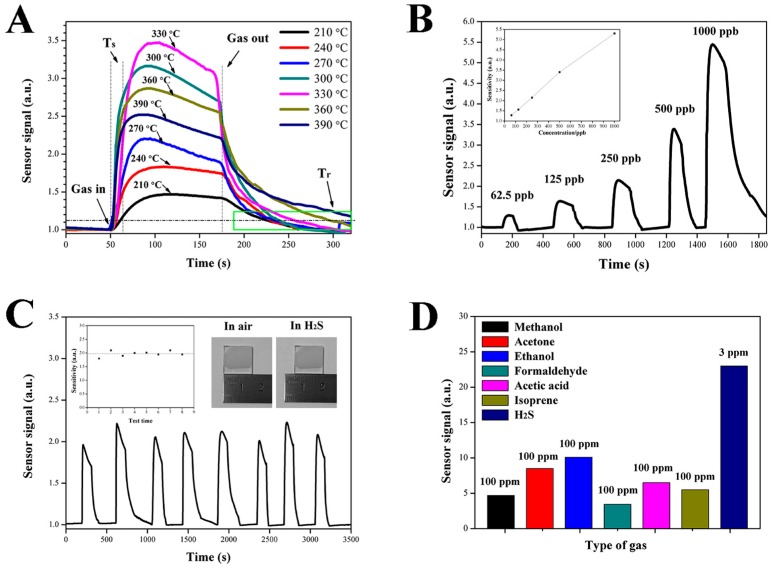
(**A**) Response of ZnO NCs sensors to 500 ppb H_2_S at a different temperature. (**B**) The response of ZnO NCs sensors to different concentrations of H_2_S at 330 °C. (**C**) Stability tests of ZnO NCs sensors to 200 ppb H_2_S at 330 °C. (**D**) Selectivity nature of ZnO NCs sensors to the different target gas.

**Figure 6:**
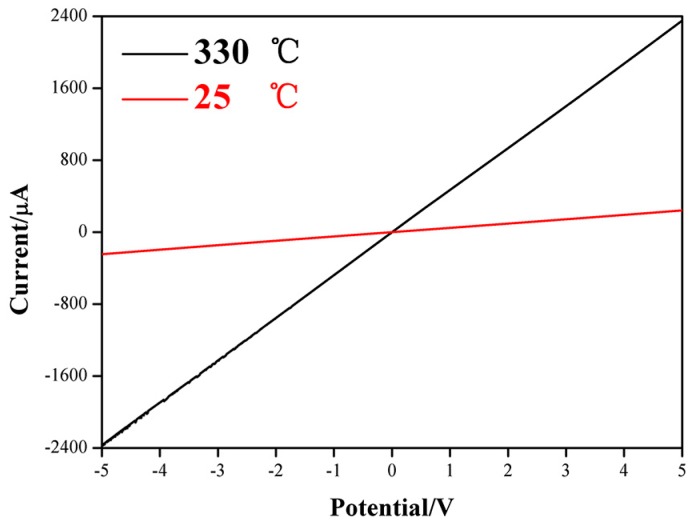
*I*-*V* characteristics measured when the ZnO NCs gas sensor is exposed to air under room-temperature and 330 °C.

The gas-sensing characteristics of the ZnO NCs gas sensor were measured under different atmospheres at different temperatures, as shown in Figure 5. Figure 5A shows the sensitivities of ZnO NCs sensor to 500 ppb H_2_S at seven temperatures from 210 °C to 390 °C. Obviously, the sensor signal of the ZnO NCs gas sensor first increases and then sharply decreases with the increase of operating temperature, and the ZnO NCs gas sensor exhibits the maximal sensor signal with a value of 3.3 at 330 °C, which indicates the optimal operating temperature of H_2_S is 330 °C. Furthermore, the impact of temperature on the response and recovery time of the ZnO NCs gas sensor has also been studied. The response time (T_s_) is defined as the time elapsed from the injection of gas (gas in) to the 90% of the maximum sensor signal, and the recovery time (T_r_) is defined as the time elapsed from the extraction of gas (gas out) to a sensor signal value of 1.11 (R_g_/R_a_ = 90%). It can be seen that the ZnO NCs gas sensor has a fast response speed with a response time of less than 10 s. For the recovery characteristics, at temperatures below 300 °C, the ZnO NCs gas sensor has similar recovery times of approximately 35 s. However, when the temperature is above 300 °C, the recovery time increases with the increase of temperature, which may be due to the fact that ZnO and H_2_S react directly to form ZnS and this reaction gradually becomes dominant as the temperature increases. The sensitivity of the ZnO NCs gas sensor to the different concentrations of H_2_S gas was tested at 330 °C, as shown in Figure 5B. It can be seen that the ZnO NCs gas sensor exhibits a remarkable response to H_2_S at very low concentrations from 62.5 to 1000 ppb. The response values for 62.5, 125, 250, 500, 1000 ppb H_2_S gas are 1.3, 1.6, 2.2, 3.3, and 5.3, respectively. The good linear tendency in the range can be clearly seen in the inset, which will be beneficial for being quantified in the practical application. Figure 6 shows the stability tests of the ZnO NCs gas sensor to 250 ppb H_2_S at 330 °C. It can be seen that the sensitivity has been maintained at around 2 for 8 consecutive gas-sensing tests. The good sensitivity should be attributed to the perfect ohmic contact between ZnO NCs and the FTO electrode and the excellent chemical stability of the FTO electrode. It can be indirectly proved that the ZnO NCs gas sensor did not produce any changes before and after the H_2_S gas-sensing tests (see the inset of Figure 5C). In addition, the ZnO NCs gas sensor was tested in the other various volatile gases, such as methanol, acetone, ethanol, formaldehyde, acetic acid and isoprene at a concentration of 100 ppm (Figure 5D). Obviously, the response to 3 ppm H_2_S is significantly higher than that of 100 ppm other gases, which indicates that the ZnO NCs gas sensor has good selectivity for H_2_S gas.

The current-voltage (*I-V*) characteristics of the ZnO NCs gas sensor were measured under room temperature (25 °C) and 330 °C, as shown in Figure 6. It can be seen clearly that a perfectly straight line is observed at each temperature, which means ZnO NCs have an ohmic contact with the FTO electrode. Based on the good contact, the response signal of ZnO NCs can be maximized due to the negligible contact resistance at the interface. Therefore, this structure that ZnO NCs in-situ grow on FTO electrode is very advantageous for enhancing the gas-sensing performances. Furthermore, the good contact between ZnO NCs and FTO electrode also will be in favor of maintaining the practical stability of gas sensor, which can be verified by the further stability test.

**Figure 7:**
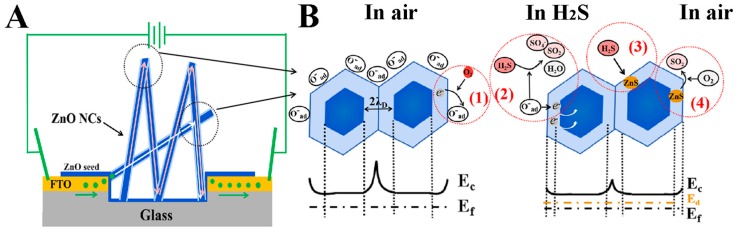
(**A**) Schematic diagrams of the electron transport path of the ZnO NCs gas sensor. (**B**) The model and band diagrams of ZnO NCs sensors exposing in air and H_2_S.

The physical mechanism of high-sensing performances of the ZnO NCs gas sensor for H_2_S gas was further investigated, as depicted in Figure 7. Figure 7A displays the electron transport paths of the ZnO NCs gas sensor. Because the ZnO NCs gas sensor mainly consists of a FTO conductive electrode and a ZnO NCs gas-sensing layer in the etched region, and while the resistance of FTO is negligible due to the extremely small magnitude, thus the electron transport paths that affect the gas-sensing performances mainly concentrate on the paths formed by the cross-linked contact of ZnO nanorods in the etched region. Generally, the cross-linked structure can provide a large number of junctions as the electron transport paths, and which has three types of junction: point junction (shaped like inverted “v”), cross junction (shaped like “x”), and block junction (shaped like inverted “y”). Among them, the point junction is very advantageous for enhancing the gas sensitivity due to the longer electron transport path that can induce a larger initial resistance, while the cross and block junctions are disadvantageous for the gas sensitivity [22]. Noteworthily, most of the connection types are the point junctions at the vertices of ZnO nanorods in our work (see Figure 2), which is a main reason for the high-performance H_2_S gas sensing. Furthermore, these point junctions formed in ZnO NCs will create additional grain boundary barriers and are also advantageous for enhancing the H_2_S gas-sensing performances [23], which will be discussed in detail next.

On the basis of the electron transport path, the models and band diagrams of the ZnO NCs gas sensors are displayed in Figure 7B. In the air, the electrons on the surface of the ZnO NCs will transfer to oxygen molecules and form several types of chemisorbed oxygen species (O^−^, O^2−^, and O_2_^−^) [24], since the conduction band edge of ZnO (about 4.3 eV below the vacuum energy) is higher than the chemical potential of O_2_ (about 5.7 eV below the vacuum energy) [25]. At this time, the ZnO surface generates a depletion layer of low electron density (resistance rise) and the conduction band level is bent upward (barrier). Especially at the junctions, a higher/wider barrier is generated, causing electrons to transfer through one rod to the other through the tunneling effect. Thus, compared to single ZnO nanorods, ZnO NCs with many point junctions will possess larger resistance due to that the probability of tunneling is inversely proportional to the height/thickness of potential barrier [26]. This process can be described by Equations (1)–(4) [24]:

(1) $$O_{2}(g)\to O_{2}(ads)$$

(2) $$O_{2}(ads){+e}^{-}\to O_{2}^{-}(ads)\;<373\;K$$

(3) $$O_{2}^{-}(ads)+2e^{-}\to 2O^{-}(ads)\;373\;K\text{–}573\;K$$

(4) $$2O^{-}(ads){+e}^{-}\to O^{2-}(ads)\;>573\;K$$

When the ZnO NCs sensor is transferred to a H_2_S atmosphere, the chemisorbed oxygen species react with H_2_S and return electrons to ZnO NCs, resulting in a decrease in the potential barrier and an increase in the electron density (resistance decline), which can be explained by Equations (5) and (6) [1,27,28,29]:

(5) $$H_{2}S(g)+2O_{}^{-}(ads)\to H_{2}(g)+SO_{2}(g)+2e^{-}$$

(6) $$H_{2}{S(g)+2O}^{2-}(\mathrm{ads})\to H_{2}(g){+\mathrm{SO}}_{4}^{-}{+e}^{-}$$

Remarkably, the change in barrier height is sensitive to gas changes and can be used as an additional signal to account for gas-sensing reactions. The behavior of the barrier controlling the transfer of charge at the junction can be expressed as [25]:

$$R=R_{0}\exp\{-e\Delta V_{b}/k_{B}T\},$$

∆V_b_ is the change of potential barriers in the air and H_2_S atmosphere, *e* the charge of an electron, T the absolute temperature, k_B_ the Boltzmann constant. And according to Feng et al. [25], the barrier model is well suitable and dominates for the detection of low concentrations of gases.

Moreover, in previous reports, the high sensitivity to H_2_S gas is also attributed to another reaction. When the operating temperature is above 300 °C, the direct reaction of H_2_S and ZnO NCs serves as the dominant sensitive mechanism, which can be expressed as Equation (7) [30]:

(7) $$ZnO(s)+H_{2}S(g)=ZnS(s)+H_{2}O(g).$$

H_2_S enters the bulk ZnO and forms zinc sulfide (ZnS). The chemisorbed S tends to trap oxygen vacancies to form shallow donor levels. Therefore, when thermally excited, electrons transition from the shallow donor level to the conduction band of ZnO, further reducing the resistance and increasing the sensitivity.

The corresponding desorption process [31,32] for this reaction can be described by Equation (8):

(8) $$2ZnS(s)+O_{2}(g)=2ZnO(s)+2SO_{2}(g).$$

ZnS reacts with oxygen in the air and returns to ZnO. Remarkably, the process of restoring the resistance to the initial value requires two processes of desorption of ZnS and adsorption of O_2_, which results in longer recovery time.

## 4. Conclusions

In summary, we have in-situ prepared ZnO NCs arrays on the etched FTO glass as a low-cost and high-performance gas sensor by the facile dip-coating and hydrothermal methods. ZnO NCs array exhibits uniform morphology, pure crystalline phase, high crystallinity and good contact with FTO electrodes. The gas-sensing measurement reveals that the ZnO NCs gas sensors show a significant response to the low-concentration H_2_S gas. The high-performance sensing can be explained by the following three aspects: (1) in-situ grown ZnO NCs have a large specific surface area compared to ZnO films obtained by the blade coating method; (2) the perfect ohmic contact between ZnO NCs and FTO electrode maximizes the response signals of ZnO NCs; (3) the variation of grain boundary barrier at the junctions of multiple nanorods can process additional gas-sensitive signals compared to the single nanorod. The low-cost, high sensitivity and excellent stability will make the ZnO NCs gas sensors have wide potential applications. We believe that this structure can facilitate the development of an inexpensive, high-performance gas sensor.
